# Anomalous enhancement of the sheet carrier density beyond the classic limit on a SrTiO_3_ surface

**DOI:** 10.1038/srep25789

**Published:** 2016-05-12

**Authors:** Neeraj Kumar, Ai Kitoh, Isao H. Inoue

**Affiliations:** 1 National Institute of Advanced Industrial Science and Technology (AIST), Tsukuba 305-8565, Japan

## Abstract

Electrostatic carrier accumulation on an insulating (100) surface of SrTiO_3_ by fabricating a field effect transistor with Parylene-C (6 nm)/HfO_2_ (20 nm) bilayer gate insulator has revealed a mystifying phenomenon: sheet carrier density 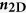 is about 10 times as large as 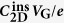 (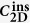 is the sheet capacitance of the gate insulator, *V*_G_ is the gate voltage, and *e* is the elementary charge). The channel is so clean to exhibit small subthreshod swing of 170 mV/decade and large mobility of 11 cm^2^/Vs for 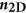 of 1 × 10^14^ cm^−2^ at room temperature. Since 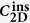 does not depend on either *V*_G_ nor time duration, 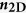 beyond 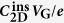 is solely ascribed to negative charge compressibility of the carriers, which was in general considered as due to exchange interactions among electrons in the small 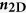 limit. However, the observed 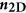 is too large to be naively understood by the framework. Alternative ideas are proposed in this work.

The Gauss’s law *Q* = *CV* in a field effect transistor (FET) is generally believed to be 
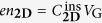
, where, *e*, *n*_2D_, 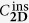, and *V*_G_ are the elementary charge, sheet carrier density of the channel, sheet capacitance of the gate insulator, and gate voltage, respectively^1^. The equation is valid, but only when the channel is an ideal metal, where the gate electric field is completely screened (zero screening length) at the channel surface due to the infinite charge compressibility 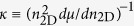 (*μ* is the chemical potential). Meanwhile, for finite *κ*, 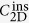 is replaced by 
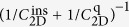
. 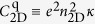 is called a quantum capacitance^2^. Total energy of the carriers corresponds to 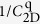, thus, in general, 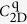 is positive and 
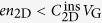
. Nevertheless, negative *κ*, for which 
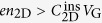
, is a long-standing target of research both experimentally^3^,^4^,^5^,^6^,^7^,^8^,^9^ and theoretically^10^,^11^, manifesting itself due to strong exchange interactions between carriers. Especially, in two-dimensional electron system (2DES), the exchange energy is negative and scales as 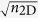, while the positive (*e.g.*, kinetic) energy scales as *n*_2D_; therefore, for sufficiently small *n*_2D_, the total energy 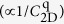 can be negative. What we demonstrate here is, however, far beyond the classic examples. A quasi-2DES at the channel of SrTiO_3_ FET shows anomalous enhancement of *n*_2D_: ten times as large as 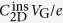. The enhancement cannot be explained only by the exchange interaction, suggesting another mechanism of inducing negative *κ*.

A schematic cross-section of a standard FET is shown in Fig. 1 with the band diagrams and the relationships between the capacitances following a widely-accepted concept of the accumulation-type metal-oxide-semiconductor FET; thick substrate (channel) of the FET is implicitly grounded in the far distance, which gives zero of the chemical potential. The gate voltage 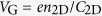 is a sum of the voltage drop in the gate insulator 
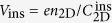
 and the band-bending of the channel material 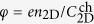. Therefore, *V*_G_ = *V*_ins_ + *φ* means 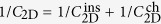 (Fig. 1a). For the metallic channel, the chemical potential (Fermi energy) *μ*/*e* substitutes for *φ*, and 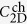 is replaced by the quantum capacitance 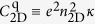 (Fig. 1b). It is still possible to consider 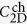 for the nonmetallic bulk part of the substrate, and 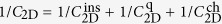. But 
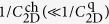
 term is usually omitted. The channel material in this study is SrTiO_3_. It changes from an insulator to metal by gating^12^, so we use a notation 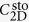 in lieu of both 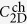 and 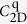. SrTiO_3_ is a classic material for solid-state physics but is a cynosure of modern oxide-electronics researches because of the formation of quasi 2DES at the surface^13^,^14^,^15^ or interface^16^,^17^, as well as the large mobility of the confined 2D carriers without a freeze-out^18^,^19^,^20^,^21^. Both the confinement and the large mobility are originated in or, if not more, influenced by the quantum paraelectricity^22^ with a large and nonlinear dielectric response^23^. Furthermore, at the surface and interface, where the inversion symmetry is broken, the charge confinement induces some intriguing electronic properties; for example, the Rashba spin-orbit coupling at the surface of SrTiO_3_ discussed in refs 24, 25, 26.

However, it is intensely difficult to fabricate such a high quality FET on SrTiO_3_ as to reveal the true nature of the exotic phenomena. The band gap of SrTiO_3_ is nearly 3.2 eV^27^,^28^, but it turns to be a good metal by *a very tiny electron doping* of 8.5 × 10^15^ cm^−3^ (corresponding to the removal of a few oxygen atoms out of 10^7^), which is orders of magnitude lower than the threshold of metallicity in Si (3.5 × 10^18^ cm^−3^) or Ge (3.5 × 10^17^ cm^−3^)^29^. Thus, the channel of SrTiO_3_ FET becomes conductive quite easily by the oxygen-defect formation. In other words, the channel current of some SrTiO_3_ FETs might be rather dominated by electrochemical reaction than purely electrostatic carrier-density modulation^30^. Therefore, in this paper, we propose an alternative gate insulator: an organic/inorganic bilayer consisting of 6 nm ultra-thin poly-monochloro-*para*-xylylene (Parylene-C) and 20 nm HfO_2_, as schematically shown in Fig. 2a. The film of Parylene-C polymer is widely used for coating a variety of material surfaces, because it is highly conformal, pin-hole free, quite inert to any gases and chemicals, and sufficiently stable from around 200 °C down to at least 60 mK^31^. The bilayer gate insulator was deposited on the atomically-flat (100) surface (miscut-angle is less than about 0.03°) of non-doped SrTiO_3_ single crystals provided by Shinkosha Co., Ltd. The photos of our FETs are shown in Fig. 2, and the cross-section images obtained by the transmission electron microscopy (TEM) are shown in Fig. 3a–d. The step and terrace surface of our SrTiO_3_ crystals is noticeably insulating with the sheet resistance above our instrumental limit (~10^13^ Ω) at room temperature, and this surface is kept sufficiently insulating after the fabrication process of FET.

The high quality of our FET manifested itself in the subthreshold behaviour. Figure 3e shows *I*_SD_ − *V*_SD_ plot for fixed *V*_G_ for the three-terminal device (See Fig. 2c). *I*_SD_ ∝ *V*_SD_ for small *V*_G_, but it shows upward convex for 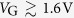, which is called the threshold voltage *V*_th_ and the region *V*_SD_ < *V*_th_ is dubbed as the subthreshold region. As shown in the *I*_SD_ − *V*_G_ curves in Fig. S1 of the Supplementary Materials, it is seen in the subthreshold region that log_10_ *I*_SD_ ∝ *V*_G_. Indeed, with *φ* in Fig. 1a, *I*_SD_ ∝ exp(*eφ*/*k*_B_*T*), where *T* is the temperature and *k*_B_ is the Boltzmann constant, because only the thermally activated carriers of the valence band can contribute to the transport. Since 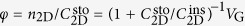, the subthreshold swing defined as 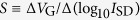 can be expressed as 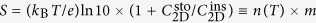. *n*(*T*) is denoted as transport factor, and *m* as body factor^32^,^33^. By measuring *S*, we can obtain 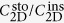 ratio.

*S* is in general estimated from the *I*_SD_ − *V*_G_ plot, but it might include a significant contribution of the contact resistance. Therefore, we deduced *S* in a more comprehensive manner. We carried out *I*_SD_ − *V*_G_ measurements in the subthreshold region with fixed *V*_SD_ = 1 V for the devices with *L* = 2, 4 and 9 *μ*m and *W* = 4*L*. Then, *WR*_*exp*_ was plotted against *L* (Fig. 3f). Here, *R*_*exp*_ = *V*_SD_/*I*_SD_ at 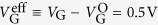. 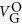 is the cut-off gate voltage, below which *I*_SD_ is smaller than the noise level of 100 fA (see Supplementary Materials for details). By comparing *WR*_*exp*_ with *R*_O_ + *LR*_2D_, the sheet resistance *R*_2D_ is obtained. We applied this method for dozens of 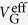, and all the *R*_2D_ values were plotted in Fig. 3g. We can see log_10_ *R*_2D_ is clearly proportional to 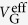, and deduced *S* = 171 mV/decade. This is indeed in good coincidence with the values simply estimated from the *I*_SD_ − *V*_G_ plot, indicating that the contact resistance of our FET does not contribute to the *S* value. Since the material-independent transport factor *n*(*T*) = (*k*_B_ *T*/*e*)ln10 is 60 mV/decade at 300 K, *S* = 171 mV/decade of this study is surprisingly small. (It was reported that the value of *S* was ~100 mV/decade even for Si^34^, ~250 mV/decade for SrTiO_3_^35^, and ~1200 mV/decade for KTaO_3_^36^). From *m* = 2.8, we deduced 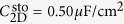. If we assume the dielectric constant of SrTiO_3_ is 310 at room temperature, the effective thickness for 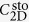 is 0.55 *μ*m. This means in the subthreshold region the gate electric field can penetrate into deep bulk of SrTiO_3_ (0.55 *μ*m) without a large Thomas-Fermi screening of free carriers possibly originated in the defects of Parylene-C/SrTiO_3_ interface. Put plainly, the Parylene-C passivation on the defect-prone SrTiO_3_ surface^37^,^38^ works fairly well. This is one of the two important premisses of this study.

The other premise is that the ultra-thin Parylene-C film works not only as a passivation layer protecting SrTiO_3_ channel from the high-*k* dielectric HfO_2_ but also works as a good capacitive layer by itself. We fabricated Ti(10 nm)/Parylene-C (3 nm)/HfO_2_ (20 nm)/Ti(5 nm)/Au(500 nm) parallel plate capacitors, and scrutinised the capacitance by both quasi-static and ac measurement. Details are given in the Supplementary Materials. The deduced sheet capacitance of the gate insulator of our FET, Parylene-C (6 nm)/HfO_2_ (20 nm), is 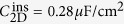 as well as the dielectric constants of 21.5 and 2.70 for the HfO_2_ layer and the Parylene-C layer, respectively, consistent to the values of 20 and 3.15 reported in literature. Alternatively, we may also assume the dielectric constants of 20 and 3.15 for HfO_2_ and Parylene-C, respectively. Then, the film thickness becomes 18.6 nm and 3.5 nm for HfO_2_ and Parylene-C, respectively, both of which are almost equivalent to the results of TEM.

By using this bilayer gate insulator, we have finally obtained both the fairly clean channel and the continuous electrostatic control of the carrier density on SrTiO_3_. This achievement, however, has given a new twist to the research of SrTiO_3_. Figure 4a shows *n*_2D_ obtained by the Hall effect measurement for the multi-terminal FET device (Fig. 2d: details of the experiments are described in the Supplementary Materials). As mentioned above, *n*_2D_ = *C*_2D_ (*V*_G_ − 1.88)/*e*, where 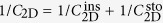 with 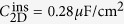 and 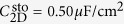. Thus, *n*_2D_ = 1.1 × 10^12^(*V*_G_ − 1.88) cm^−2^. (It should be noted here that the 1.88 V offset, above which the accumulation of the carriers in the channel becomes observable by the Hall effect measurements, may be due to the relatively larger contact resistance of the multi-terminal FET device used for the measurements; however, the origin of this offset does not affect to the following discussion). To our surprise, the measured *n*_2D_ is *much larger than this naive estimation*; it reaches to around 1 × 10^14^ cm^−2^ for *V*_G_ = 6 V. Even if this extra carriers are provided by the formation of oxygen/cation defects in the SrTiO_3_ channel during the application of the large *V*_G_ (though the channel is fairly protected by Parylene-C layer and is actually clean), it should be noted that *n*_2D_ cannot be modulated without a change of *C*_2D_, independent of sources of the carriers.

We have measured the Hall effect for more than ten FET devices on three different SrTiO_3_ substrates (two results are shown in Fig. S3), and confirmed all of them showed qualitatively same *n*_2D_ enhancement. In order to explain this large discrepancy, we have assumed a naive model that the channel is a *serial* connection of a bulk SrTiO_3_ (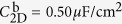), and a surface layer 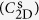. When *V*_G_ is small, 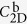 is most dominant to 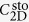, but as *V*_G_ increased, accumulated carriers screen the gate voltage; *i.e.*, for *V*_G_ > *V*_min_, 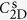 becomes more dominant. Then, we introduced a tractable model: 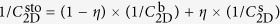, where 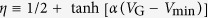. (*α* = 0.68 and *V*_min_ = 2.8 V are non-essential parameters). This is an ad-hoc phenomenological model to express that 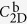 changes smoothly from 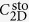-dominant to 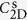-dominant, thus the mathematical formula is not relevant. If 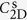 is a large positive number as that of a good metal, 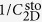 and corresponding *n*_2D_ behave as dash-dotted lines (purple) in Fig. 4a. Deviation is still large. Then, if we assume negative capacitance 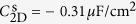, the calculated *n*_2D_ coincides with the measured *n*_2D_.

We understand that *I*_SD_, 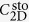 and *n*_2D_ should behave as shown schematically in Fig. 4b. Negative 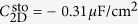, *i.e.*, *negative κ*, *is inevitable for explaining the large enhancement of n*_2D_. But a question arises. If this is ascribed to the exchange interaction of the quasi-2DES on SrTiO_3_ as explained in literature^3^,^4^,^5^, averaged distance between the electrons should be much larger than the Bohr radius *a*_B_, *i.e.*, 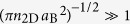, and the system may become like the Wigner crystal with negative chemical potential 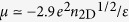, where *ε* is a direlectric constant of SrTiO_3_ (ref. 39). However, *n*_2D_ in this study is in the order of 10^14^ cm^−2^, then the corresponding values 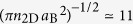 and 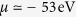 are both unreasonable. It was suggested that negative *κ* is also realised in electronic systems close to half filling^40^, but this neither is applicable to our samples. Therefore, the significant enhancement of *n*_2D_ cannot be explained solely by the negative *κ* originating in the exchange interactions; we need an alternative idea.

Then, we consider the shift of *μ* further. In a rigid-band model, where the binding energy of each band shifts monotonously without changing the gaps, *μ* increases by the electron doping and decreases by the hole doping, always leading to positive *dμ*/*dn*_2D_ and thus positive *κ* as shown in Fig. 4c (top). On the contrary, in strongly correlated electron systems, the carrier doping drives the spectral weight transfer (naively a change of the density of states) from the higher energy incoherent states to the lower energy quasiparticle band to fill the Mott-Hubbard gap. Since the band gap decreases, *μ* decreases effectively and *dμ*/*dn*_2D_ becomes negative^41^ as shown in Fig. 4c (middle) more interesting is that the carrier confinement at the surface of SrTiO_3_ with perpendicular gate electric field gives rise to the Rashba effect^42^. If the Rashba spin-orbit coupling is large, the band structure depends on the gate voltage, *i.e.*, *n*_2D_, leading to a non-rigid band structure as well. That is, the coupling lowers the band edge quadratically, and thus the negative *dμ*/*dn*_2D_ is realised^43^,^44^ as depicted in Fig. 4c (bottom). However, the absolute value of our negative capacitance −0.31 *μ*F/cm^2^, which corresponds to *dμ*/*dn*_2D_ = −5.1 × 10^−13^ eV cm^2^, is too large. For *V*_G_ between 4 V and 6 V, Δ*n*_2D_ is around 5 × 10^13^ cm^−2^, then 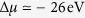, which is difficult to be understood either by the Mott transition^10^,^41^ or the Rashba effect^43^,^44^,^45^.

We think a clue to approach this problem is an inhomogeneity of the channel. As shown in Fig. S7c in the Supplementary Materials, we have observed a sudden decrease of the internal voltage distribution in the channel along *I*_SD_ while increasing *V*_G_. This has already been observed in other SrTiO_3_ -FET, indicating a formation of conducting domains in the insulating matrix, which eventually forms a conducting filament by percolation^19^. Here we assume that the channel consists of two regions, *i.e.*, the metallic domains with the negative sheet capacitance 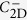, and the non-metallic matrix with the normal positive sheet capacitance 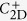. Then, the channel sheet capacitance 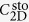, which is given by 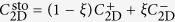 with the volume fraction *ξ* can be −0.31 *μ*F/cm^2^, even if 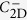 is the value which gives a reasonably small Δ*μ*. Details are given in the Supplementary Materials.

Inhomogeneity of Parylene-C thickness in our gate insulator (~30% at most), and other features such as the one dimensional metallic state at the step edge of SrTiO_3_ (ref. 46) would be the origins of charge inhomogeneity. Moreover, the large positive *κ*, which the insulating SrTiO_3_ substrate holds due to the quantum paraelectricity, can augment the inhomogeneities further. Nevertheless, those “*extrinsic inhomogeneities*” cannot explain the 1000% enhancement of *n*_2D_ as observed in this study. Therefore, we made an inference that an electronic phase separation with the spinodal instability may be induced by *κ* → 0, *i.e.*, 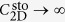 in our case^47^. In fact, the phase separation and the charge segregation are natural consequences of the negative capacitance even in ideally homogeneous 2DES^43^. The charge segregation may cause a local charge imbalance at finite length scales. The frustration between the electrostatic cost and the energy gain due to the phase separation is a possible mechanism of charge inhomogeneous (stripe) states^48^. However, in our FET, the local charge on the SrTiO_3_ surface is balanced by the charge on the gate, thus the frustration may be weaken and the typical size of inhomogeneous regions can be microscopic. We hope that this insight motivate further investigation and brings us better understanding of the intriguing physics still hidden in the SrTiO_3_ surface.

In summary, *n*_2D_ of the channel of SrTiO_3_ FET with Parylene-C (6 nm)/HfO_2_ (20 nm) hybrid gate insulator showed anomalous enhancement: ten times as large as the expected value 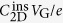, indicating negative *κ*, *i.e.*, negative 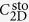. However, if the whole channel is a single metallic state with the negative *κ*, the chemical potential shift becomes too large. On the other hand, transport behaviour suggests the inhomogeneous carrier distribution of the channel, though the channel is fairly clean as evidenced by the small subthreshold swing *S* = 171 mV/decade and large carrier mobility ~11 cm^2^/Vs. An intrinsic electronic inhomogeneity is a natural consequence of the negative *κ*, thus it can happen on the channel of our SrTiO_3_ -FET. The missing link among the huge *n*_2D_ enhancement, the negative *κ*, and the intrinsic inhomogeneity will be elucidated by detailed studies.

## Methods Summary

Experimental and data analysis methods with associated references are available in the Supplementary Materials.

## Additional Information

**How to cite this article**: Kumar, N. *et al*. Anomalous enhancement of the sheet carrier density beyond the classic limit on a SrTiO_3_ surface. *Sci. Rep.* **6**, 25789; doi: 10.1038/srep25789 (2016).

## Supplementary Material

Supplementary Information

## Figures and Tables

**Figure 1:**
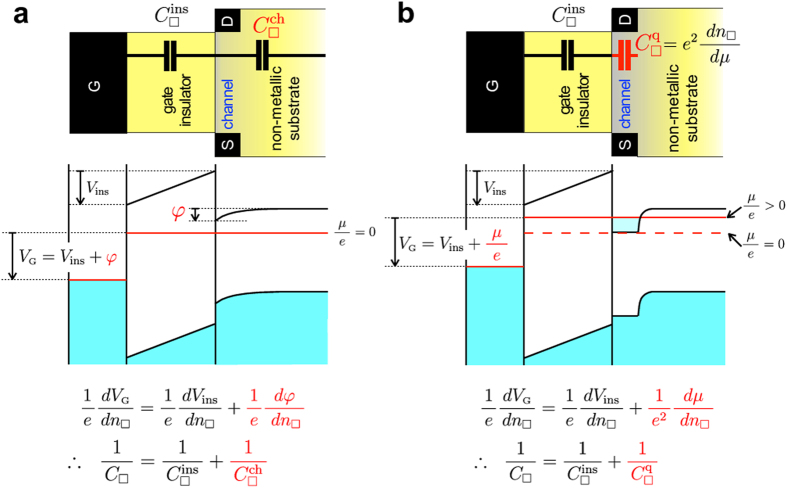
Schematic pictures of the cross section and the band diagram of FET. Neither distance nor energy of the picture scales to that of the real device. (**a**) The channel is an n-type non-metallic material (*e.g.*, non-doped SrTiO_3_). By differentiating *V*_G_ = *V*_ins_ + *φ* with respect to *n*_2D_, and by using the Gauss’s law, we obtain 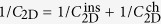. (**b**) For larger *V*_G_, the channel becomes metallic and *V*_G_ = *V*_ins_ + *μ*/*e*. Same as (**a**) the relationship 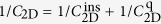 is obtained, where 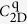 is called a quantum capacitance (ref. 2). 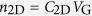 becomes larger than 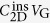 only when 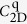 is negative.

**Figure 2:**
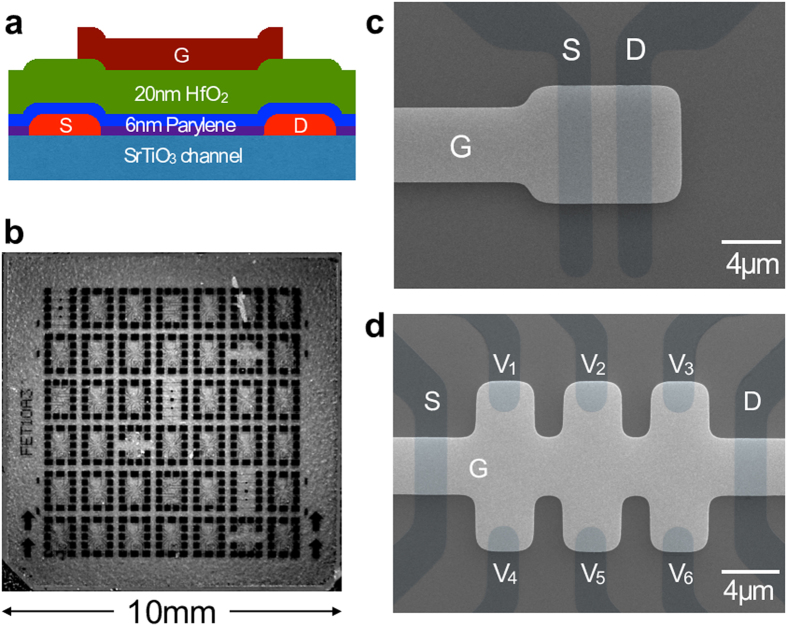
FET with HfO_2_ (20 nm)/Parylene-C (6 nm) solid-state bilayer gate insulator studied in this work. (**a**) Schematic cross-section image of our three-terminal FET device. (**b**) Photograph of a 10 mm × 10 mm (100) SrTiO_3_ substrate with the FET devices fabricated on it. Scanning electron microscopy images of (**c**) a three-terminal FET device, and (**d**) multi-terminal one. G, S and D stand for gate, source, and drain electrodes, respectively. V_1_–V_6_ are potential probes.

**Figure 3:**
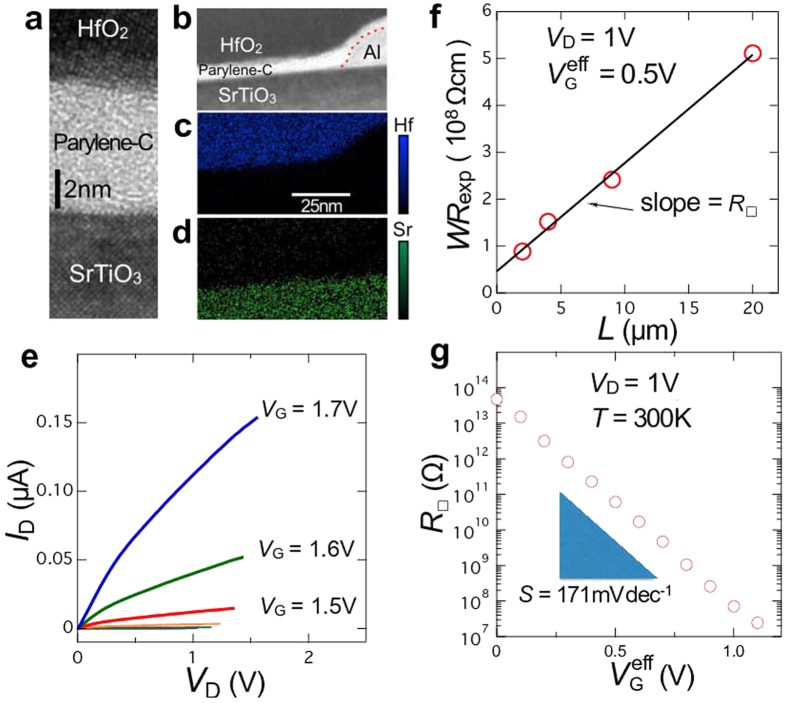
Characteristics of SrTiO_3_ FET with HfO_2_ (20 nm)/Parylene-C (6 nm) bilayer gate insulator. (**a**) Cross sectional TEM image of the channel. (**b**) Cross sectional scanning TEM (STEM) image near the Al electrode (dotted line is a guide to eyes separating Parylene-C and Al). (**c**) Energy-dispersive x-ray spectroscopy mapping for Hf atom and (**d**) that for Sr atom. (**e**) *I*_SD_ − *V*_SD_ plots for 3-terminal device with *L* = 20 *μ*m and *W* = 80 *μ*m for several *V*_G_. (**f**) 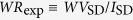 for four FETs with different sizes but fixed *W*/*L* ratio plotted as a function of *L* (open circles). Solid line is the least-square fit (*WR*_exp_ = *R*_O_ + *LR*_2D_) to deduce *R*_2D_. (**g**) *R*_2D_ vs. 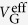 plot gives *S* of 171 mV/decade.

**Figure 4:**
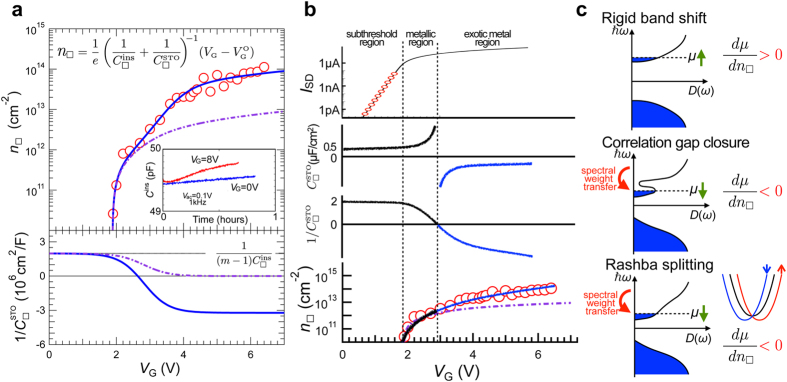
Interpretation of 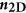 enhancement by a negative *κ* model. (**a**) Top: sheet carrier density *n*_2D_ (open circles) obtained by the Hall effect measurement for the multi-terminal FET device. Solid line (blue) is a least-square fit of the data to 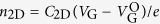, where 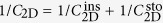, 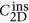 is 0.28 *μ*F/cm^2^, and 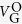 is 1.88 V. For 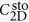, we used a model shown in the bottom panel (See main text for details). Bottom: the dash-dotted line (purple) represents a case that 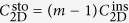 in the subthreshold region with the body factor *m* of 2.8 changes to 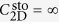 of the ideal metal. The solid line (blue) becomes negative which explains the enhancement of *n*_2D_. Inset shows the capacitance of the HfO_2_/Parylene-C gate insulator as a function of time measured while continuously applying the voltage. The variation is less than 2% for one hour even for the application of 8 V which is close to the breakdown voltage. (**b**) Schematic picture of *I*_SD_, 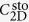 and *n*_2D_ with respect to *V*_G_. In the metal region, 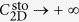, and comes back from −∞. However, 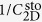 changes continuously, which explains the observed *n*_2D_. (**c**) Negative capacitance means the charge compressibility 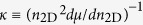 is negative, *i.e.*, *dμ*/*dn*_2D_ is negative. In the general rigid-band model, *dμ*/*dn*_2D_ > 0. If the density of states *D*(*ω*) is changed by the carrier doping, *dμ*/*dn*_2D_ < 0 can be realised. Closure of the correlation gap such as the Mott transition, and a band-splitting such as the Rashba effect are the typical examples.
