# Supplementary material for: Anomalous enhancement of the sheet carrier density beyond the classic limit on a SrTiO3 surface
Source: Sci Rep. 2016 May 12;6:25789. doi: 10.1038/srep25789 (PMC4865841; doi:10.1038/srep25789)
Supplement: Supplementary Information [file srep25789-s1.pdf]

Supplementary Material

Anomalous enhancement of the sheet carrier density beyond the classic limit on a $SrTiO_3$ surface.

By *Neeraj Kumar, Ai Kitoh,* and *Isao H. Inoue*

SAMPLE FABRICATION

Step and terrace (100) surfaces of $10 \times 10 \times 0.5\,\mathrm{mm}^3$ insulating $SrTiO_3$ single crystals having a miscut angle of less than 0.03° (Shinkosha Co., Ltd.) was used for the channel of our field-effect transistor (FET). First of all, the pristine surface of the $SrTiO_3$ single crystal was coated with a 3 nm Parylene-C layer using the Specialty Coating Systems LabCoater2 (model PDS2010). The Parylene-C film was formed according to the Gorham method:[1] the dimer was vaporised at 135 °C, decomposed into the monomer in the furnace heated to 690 °C, and then deposited at room temperature on the surface of $SrTiO_3$ placed in a deposition chamber evacuated to less than 5 mTorr. The thickness was checked briefly by the Filmetrics F20-UV film thickness monitor. Some samples were further examined by the transmission electron microscopy (TEM).

The Ultratech UTS-1700 stepper was used for the projection photolithography of the electrode pattern. To facilitate the electric contacts between the source/drain electrodes and the $SrTiO_3$ surface, Parylene-C film was selectively etched by ozone plasma under ultraviolet radiation.[2] We used 10 nm-thick Al or Ti metal for source/drain electrodes. The Al metal was evaporated by resistive heating of alumina crucible, while the Ti metal by electron beam heating (evaporation rates are 1 nm/s for Al and 0.1 nm/s for Ti). All the FET properties shown in the study do not depend on whether the source/drain electrodes may be Al or Ti. With the Shin-Etsu Chemical SIPR-9684-1.5 photoresist which forms an undercut structure, we could avoid the burr formation[3], even though all the source/drain electrodes were formed by the lift-off method.

After the electrode fabrication, a 3 nm-thick second layer of Parylene-C was deposited to cover the whole area. Then, a 20 nm-thick HfO_2 layer was deposited by Picosun SUNALE R-100B atomic layer deposition (ALD) system. $Hf[N(CH_3)_2]_4$ [tetrakis(dimethylamido)hafnium or TDMAH] was used as the hafnium metal precursor, which was preheated to 130 °C and was delivered to the reaction chamber, in which the sample was heated to 120 °C. Deionized water was used as the oxygen source for the 169-cycle ALD deposition. The thickness of HfO_2 film was 20 nm, which was estimated by the KLA-Tencor AlphaStep D-100 surface profiler, and some samples were more precisely examined by TEM. The gate electrodes were 5/500 nm Ti/Au (evaporation rate was 0.1 nm/s for Ti and 5 nm/s for Au) deposited by the e-beam evaporator.

The FET shown in Fig. 2c of the main text has the channel length L of either 2, 4, 9, or 20 μm, and the channel width $W = 4L$. The FET shown in Fig. 2d of the main text has L of either 20 or 50 μm, and W of either 4 or 10 μm, respectively. We also fabricated parallel plate capacitors of 100×100, 60×60, and 20×20 $\mu\mathrm{m}^2$ square electrodes to evaluate the dielectric properties of the gate insulator.

CROSS SECTION IMAGING

TEM and STEM imaging was carried out to investigate the cross-section of the channel of the multi-terminal sample shown in Fig. 2d of the main text. JEOL JEM-2100F equipped with bright field (BF) detectors and energy dispersive x-ray analyser with an accelerating voltage of 200 kV was used for the measurements. Slices for the TEM/STEM observations were prepared using a focused ion beam (FIB) system, JEOL JEM-9310FIB, operating at 5 kV and 30 kV.

TEM image in Fig. 3a of the main text specifies that each layer of $SrTiO_3$, Parylene-C, and HfO_2 is clearly distinguished. Total thickness of the bilaminar Parylene-C layer is around 6 nm. Although the devices were prepared by the standard lift-off method, scanning tunnelling electron microscope (STEM) near the edge of an Al source-drain electrode (Fig. 3b in the main text) showed that the edge has no such a burr to affect the device performance.[3] Furthermore, the energy-dispersive x-ray spectroscopy mappings for the Hf and Sr atoms (Fig. 3b and Fig. 3c in the main text, respectively) indicate that the mixture of atoms in each layer was almost negligible, providing a premise for evaluating our bilayer gate insulator.

CUT-OFF GATE VOLTAGE AND SUBTHRESHOLD SWING

All the current-voltage ($I_{\rm SD}$-$V_{\rm G}$ and $I_{\rm SD}$-$V_{\rm SD}$) measurements were performed using the Agilent Technology E5287A high-resolution source/monitor unit modules equipped on the E5270B precision measurement mainframe. For the measurements in air at room temperature, we used the Karl Süss probe station.

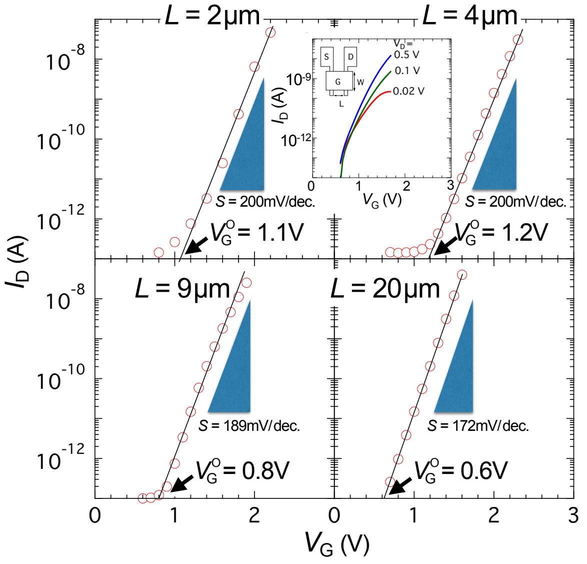

FIG. S1. Channel current $I_{\rm SD}$ in the subthreshold region for 3 terminal devices with the channel length of L = 2, 4, 9, or 20 μm, and the channel width of W=4L plotted against $V_{\rm G}$ for $V_{\rm SD}$ = 1 V (open circles). The solid lines represent the least-square fit to a model ($\log_{10} I_{\rm SD} \propto V_{\rm G}/S$) with the subthreshold swing S. Cut-off gate voltage $V_{\rm G}^{\rm O}$ below which $I_{\rm SD}$ becomes smaller than the noise level of 100 fA is defined in this way. Due to the contribution of the contact resistance, S increases by decreasing the channel length L. By subtracting the contribution of the contact resistance, as discussed in the main text, we obtained S of 171 mV/decade, which are not much different from the rough estimation here. Inset shows $I_{\rm SD}$-$V_{\rm G}$ plots for different $V_{\rm SD}$. When we decrease $V_{\rm SD}$, the subthreshold region, where $\log I_{\rm SD} \propto V_{\rm G}$, becomes smaller.

For a sufficiently reliable comparison of the $I_{\rm SD}$-$V_{\rm G}$ data of different devices, we defined an effective gate voltage $V_{\rm G}^{\rm eff}$ as $V_{\rm G}^{\rm eff} \equiv V_{\rm G} - V_{\rm G}^{\rm O}$, where $V_{\rm G}^{\rm O}$ is the cut-off gate voltage below which $I_{\rm SD}$ becomes smaller than the noise level of 100 fA. Open circles in Fig. S1 are $I_{\rm SD}$-$V_{\rm G}$ plots for $V_{\rm SD}$ = 1 V for four three-terminal FET devices with the channel length of L = 2, 4, 9, and 20 μm, and the channel width of $W = 4L$. Then, $I_{\rm SD}$-$V_{\rm G}$ data were fitted by $\log_{10}(I_{\rm SD}/100\,{\rm fA}) = (V_{\rm G} - V_{\rm G}^{\rm O})/S$ (solid lines in Fig. S1), where S is the subthreshold swing (see the main text for the definition). The fitting gave $V_{\rm G}^{\rm O}$ = 1.1, 1.2, 0.8, and 0.6 V, and S = 200, 200, 189, and 172 mV/decade for L = 2, 4, 9, and 20 μm, respectively. Using the $V_{\rm G}^{\rm eff} = V_{\rm G} - V_{\rm G}^{\rm O}$, we could safely compare the data of different L, and obtained Fig. 3c and 3d of the main text.

CAPACITANCE OF GATE INSULATOR

Agilent 4155C semiconductor parameter analyser was used for the quasi-static capacitance measurements, while a combination of the Solartron 1296 Dielectric Interface and SI 1260 impedance/gain-phase analyser was used for the ac capacitance measurements.

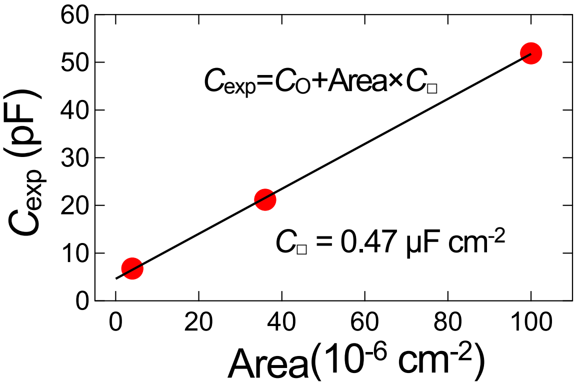

FIG. S2. Ti(10 nm)/Parylene-C(3 nm)/HfO_2(20 nm)/-Ti(5 nm)/Au(400 nm) capacitor gives capacitance $C_{\rm exp}$ proportional to the area. This is obtained by quasi-static measurement at 1 V. Sheet capacitance $C_\square^{\rm ins}$ of 0.47 μF/cm^2 is deduced.

We fabricated Ti(10 nm)/Parylene-C(3 nm)/-HfO_2(20 nm)/Ti(5 nm)/Au(500 nm) parallel plate capacitors, and measured quasi-static capacitance $C_{\rm exp}$ at a constant bias of 1 V. It should be noticed that Parylene-C for this capacitance measurement is half as thick as that in the bilayer gate insulator of our FET. As shown in Fig. S2, $C_{\rm exp}$ is proportional to the area A, and is well fitted by $C_{\rm O} + A\,C_\square^{\rm ins}$ with the parasitic capacitance $C_{\rm O}$ of 4.6 pF and the sheet capacitance $C_\square^{\rm ins}$ of 0.47 μF/cm^2. This value of $C_\square^{\rm ins}$ = 0.47 μF/cm^2 does not depend on the applied voltage. For comparison, given that the dielectric constants of HfO_2 and Parylene-C are 20 and 3.15, respectively, a serial connection of HfO_2(20 nm) and Parylene-C(3 nm) gives $C_\square^{\rm ins}$ of 0.45 μF/cm^2: very good agreement with the experimental value.

Then, in order to decompose the contributions of HfO_2 and Parylene-C layers, we measured the frequency ω dependence of $C_{\rm exp}$ for a capacitor with $A = 100 \times 100\,\mu$m^2 between 1 Hz and 1 MHz while applying a constant bias of 1 V. The results are simulated by the models shown in Fig. S3. $C_{\rm Hf}$ and $C_{\rm P}$ denote the sheet capacitance of HfO_2 and Parylene-C layers, respectively, as well as $R_{\rm Hf}$ and $R_{\rm P}$ do the resistance. Total impedance $Z(\omega)$, where $\omega \equiv 2\pi f$ and f is the frequency of the applied ac bias, is expressed as a sum of $Z_{\rm Hf}(\omega)$, $Z_{\rm P}(\omega)$, and $Z_{\rm ps}(\omega)$, which are the impedance of

HfO_2 Parylene-C and parasitic contribution, respectively. That is,

$$
\begin{aligned}
Z(\omega) &= Z_{\mathrm{P}}(\omega) + Z_{\mathrm{Hf}}(\omega) + Z_{\mathrm{ps}}(\omega) \\
&= \left(\frac{1}{R_{\mathrm{P}}} + i\omega C_{\mathrm{P}}\right)^{-1} + \left(\frac{1}{R_{\mathrm{Hf}}} + i\omega C_{\mathrm{Hf}}\right)^{-1} \\
&\quad + \left(\frac{1}{R_{\mathrm{ps}}} + i\omega C_{\mathrm{ps}}\right)^{-1} \\
&= \frac{R_{\mathrm{P}}}{1 + i\omega\tau_{\mathrm{P}}} + \frac{R_{\mathrm{Hf}}}{1 + i\omega\tau_{\mathrm{Hf}}} + \frac{R_{\mathrm{ps}}}{1 + i\omega\tau_{\mathrm{ps}}} \ ,
\end{aligned}
$$

where C_{ps} and R_{ps} are the parasitic capacitance and resistance, as well as $\tau_{\mathrm{P}} \equiv R_{\mathrm{P}}\, C_{\mathrm{P}}$, $\tau_{\mathrm{Hf}} \equiv R_{\mathrm{Hf}}\, C_{\mathrm{Hf}}$, and $\tau_{\mathrm{ps}} \equiv R_{\mathrm{ps}}\, C_{\mathrm{ps}}$. Total capacitance $C(\omega)$ of the double layer is given by the imaginary part of $Z(\omega)$ as

$$
\begin{aligned}
\frac{1}{C(\omega)} &= -\omega\, \mathrm{Im} Z(\omega) \\
&= \frac{(\omega\tau_{\mathrm{P}})^2}{1 + (\omega\tau_{\mathrm{P}})^2}\frac{1}{C_{\mathrm{P}}} + \frac{(\omega\tau_{\mathrm{Hf}})^2}{1 + (\omega\tau_{\mathrm{Hf}})^2}\frac{1}{C_{\mathrm{Hf}}} \\
&\quad + \frac{(\omega\tau_{\mathrm{ps}})^2}{1 + (\omega\,\tau_{\mathrm{ps}})^2}\frac{1}{C_{\mathrm{ps}}} \\
&= \left(\frac{\tanh\psi_{\mathrm{P}} + 1}{2}\right)\frac{1}{C_{\mathrm{P}}} + \left(\frac{\tanh\psi_{\mathrm{Hf}} + 1}{2}\right)\frac{1}{C_{\mathrm{Hf}}} \\
&\quad + \left(\frac{\tanh\psi_{\mathrm{ps}} + 1}{2}\right)\frac{1}{C_{\mathrm{ps}}} \\
&\equiv \frac{1}{C_{\mathrm{P}}(\omega)} + \frac{1}{C_{\mathrm{Hf}}(\omega)} + \frac{1}{C_{\mathrm{ps}}(\omega)} \ ,
\end{aligned}
$$

where $\psi_{\mathrm{P}} \equiv \ln(\omega\tau_{\mathrm{P}})$, $\psi_{\mathrm{Hf}} \equiv \ln(\omega\,\tau_{\mathrm{Hf}})$, and $\psi_{\mathrm{Hf}} \equiv \ln(\omega\tau_{\mathrm{Hf}})$. It is worth noting here that each component of $1/C_\phi(\omega)$ behaves as $\tanh(\psi_\phi)$, where ϕ denotes either Hf, P or ps, as schematically shown in Fig. S3a. When we plot $1/C_\phi(\omega)$ as a function of $\log_{10}\omega$, the slope at $\psi_\phi = 0$, *i.e.*, at $\omega = 1/\tau_\phi$, is given by

$$
\begin{aligned}
&\frac{d}{d(\log_{10}\omega)}\left(\frac{1}{C_\phi(\omega)}\right)\Bigg|_{\omega=1/\tau_\phi} \\
&= \frac{1}{C_\phi}\frac{d\psi}{d(\log_{10}\omega)}\Bigg|_{\omega=1/\tau_\phi} \\
&= \frac{1}{C_\phi}\frac{1}{\log_{10}\mathrm{e}}
\end{aligned}
$$

independent of R_ϕ, where e is the base of natural logarithm. This means the width of the slope at $\omega = 1/\tau_\phi$ is fixed to $\log_{10}\mathrm{e}$, and the slow increase of measured $1/C_\phi(\omega)$ as a function of $\log_{10}\omega$ cannot be explained by this naive model. This is clearly seen in Fig. S3b.

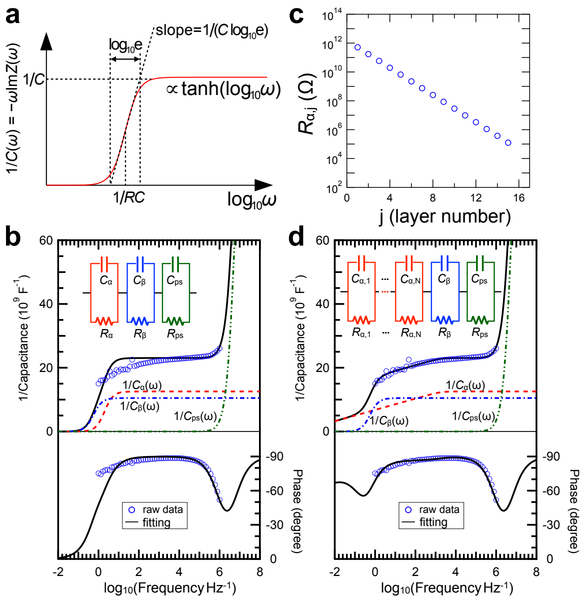

FIG. S3. **a,** $1/C(\omega) = -\omega\,\mathrm{Im}Z(\omega) = (\tanh\psi + 1)/2C$ plotted against $\log_{10}\omega$. **b,** Measured $1/C(\omega)$ (open circles) are fit to an equivalent circuit model (solid line). A schematic picture of the model shown in the inset consists of three components: $1/C_\alpha(\omega)$ (dashed line) stands for the capacitances C_α with the parallel resistance of R_α, $1/C_\beta(\omega)$ (dashed-dotted line) does C_β and R_β, and $1/\,C_{\mathrm{ps}}(\omega)$ (dashed-two dotted line) does C_{ps} and R_{ps}. $1/\,C_{\mathrm{ps}}(\omega)$ represent all the contributions except for those of Parylene-C and HfO_2, while either $1/C_\alpha(\omega)$ or $1/C_\beta(\omega)$ may correspond to either Parylene-C or HfO_2. **c,** The model in **b,** is improved by introducing multiple sublayers for the α layer shown in inset of **d,** . The resistance of each sublayer $R_{\alpha,j}$ changes from $\sim 10^{12}\,\Omega$ to $\sim 10^{5}\,\Omega$ as we change the sublayer number j from 1 to $\mathrm{N} = 15$. **d,** Same as **b,** but the model is different. See text in details.

Therefore, we assumed either HfO_2 or Parylene-C layer has a gradient of resistance along the thickness. Here the layer is divided into N sublayers, and each sublayer has a capacitance of $\mathrm{N}C_\alpha$ with a parallel resistance of $R_{\alpha,j} (j = 1, 2, ..., \mathrm{N})$. For simplicity, the slow increase of the measured $1/C_\alpha(\omega)$ is supposed to be proportional to $\log_{10}\omega$ between ω_1 and ω_{N}. In other words, $\log_{10} R_{\alpha,j}$ changes *linearly* from $\log_{10}(1/\mathrm{N}C_\alpha\omega_1)$ to $\log_{10}(1/\mathrm{N}C_\alpha\omega_{\mathrm{N}})$ (Fig. S3c). A schematic picture of the model is depicted in the inset of Fig. S3d, and the solid line of Fig. S3d demonstrates that the model can fit the measured capacitance fairly well. The values of the parameters used in the fittings are summarised in Table S1.

From the values of $C_\alpha = 79.6\,\mathrm{pF}$ and $C_\beta = 95.3\,\mathrm{pF}$ obtained from the fitting, it is reasonable to consider the subscript α denotes Parylene-C while β denotes HfO_2. Then, the dielectric constant of our HfO_2 layer becomes about 21.5, and

TABLE S1. Values of the parameters used for the fitting of ac capacitance $C(\omega)$ to an equivalent circuit model shown in Fig. S3b and Fig. S3d. C_{P}, C_{Hf}, and C_{ps} stand for the capacitances of Parylene-C HfO_2 and some parasitic contribution, respectively, while R_{P}, R_{Hf}, and R_{ps} are their resistances which are set parallel to the capacitors. For the fitting in Fig. S3d, we assumed that the linear change of $1/C(\omega)$ in the region where $\log_{10}\omega$ between $\log_{10}\omega_1$ and $\log_{10}\omega_{\mathrm{N}}$ is due to the linear change of $\log_{10}R_{\mathrm{P},j}$ from $\log_{10}R_{\mathrm{P},1}$ to $\log_{10}R_{\mathrm{P,N}}$, where $j = 1, ..., \mathrm{N}$ is the index of the N sublayers within the 3 nm Parylene-CṪhat is, $R_{\mathrm{P},1} = 1/\mathrm{N}\, C_{\mathrm{P}}\,\omega_1$ and $R_{\mathrm{P,N}} = 1/\mathrm{N}\, C_{\mathrm{P}}\,\omega_{\mathrm{N}}$

	Fig. S3b	Fig. S3d
R_{P} (GΩ)	0.504	–
N	–	15
$\log_{10}(2\pi\omega_1)$	–	−3.586
$\log_{10}(2\pi\omega_{\mathrm{N}})$	–	3.024
C_{P} (pF)	79.64	79.64
R_{Hf} (GΩ)	2.963	2.963
C_{Hf} (pF)	95.28	95.28
R_{ps} (kΩ)	3.439	3.439
C_{ps} (pF)	7.668	7.668

that of Parylene-C becomes about 2.70, both of which are quite comparable with 20 and 3.15, respectively, reported in many literatures. (On the other hand, If we assume the dielectric constant of HfO_2 is 20, its thickness becomes 18.6 nm, and that of Parylene-C with the dielectric constant of 3.15 becomes 3.5 nm, almost equivalent to the results of TEM.) The parallel resistance of the 3 nm Parylene-C layer has a variation from $10^{12}\,\Omega$ down to $10^{5}\,\Omega$ (Fig. S3c). In our naive model, we assumed the resistance may change in the film; instead, it is also possible to assume the capacitance may have a variation.[4] In reality, we think both the capacitance and the resistance may change. Since our main objective in this study was to utilise the ultra-thin Parylene-C for a passivation layer, and it was successfully confirmed. Therefore, we leave the mechanism of the resistance/capacitance variation of the ultra-thin Parylene-C film for future studies.

It should be noted in passing that the high frequency limit of the capacitance C_∞ without the parasitic contribution is

$$\begin{aligned}\frac{1}{C_\infty} &= \frac{1}{C_{\mathrm{P}}} + \frac{1}{C_{\mathrm{Hf}}}\\ &= \frac{1}{79.6\,\mathrm{pF}} + \frac{1}{95.3\,\mathrm{pF}}\\ &= 2.31\times 10^{10}\,\mathrm{F}^{-1}\end{aligned}$$

which gives $C_\square = C_\infty/(100\,\mu\mathrm{m})^2 = 0.43\,\mu\mathrm{F/cm}^2$. This value is in reasonable agreement with $0.47\,\mu\mathrm{F/cm}^2$ obtained by the quasi-static measurement described in the main text.

HALL EFFECT MEASUREMENTS

Hall effect measurements were carried out using the Agilent Technology E5287A high-resolution source/monitor unit modules equipped on the E5270B precision measurement mainframe. The

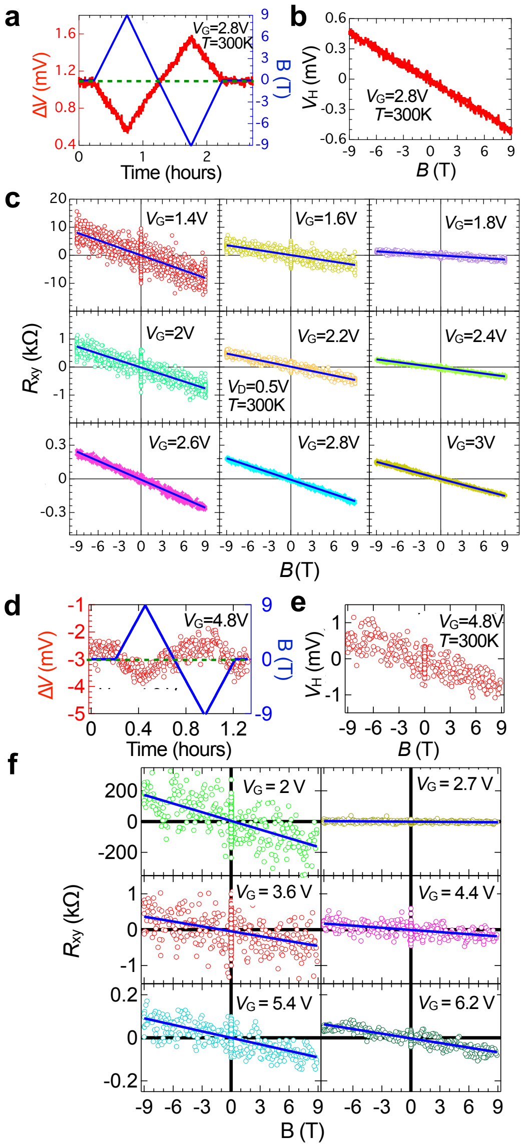

FIG. S4. **a,** Jagged solid line (red) is the voltage difference ΔV between V_2 and V_5 probes (Fig. 2d of the main text) for $V_{\mathrm{G}} = 2.8\,\mathrm{V}$ and $V_{\mathrm{SD}} = 0.5\,\mathrm{V}$, and the smooth solid line (blue) corresponds to the the magnetic field B applied perpendicular to the sample surface. Both are plotted against the elapsed time. **b,** Hall voltage V_{H} shows almost linear dependence on B. **c,** Hall resistance $R_{\mathrm{xy}} = V_{\mathrm{H}}\,/\,I_{\mathrm{SD}}$ (open circles) plotted against B for different V_{G}. The solid line in each panel is a least-squares linear fitting of R_{xy} to deduce the Hall coefficient $R_{\mathrm{H}} = R_{\mathrm{xy}}\,/B$. **d, e, f,** Same as **a, b, c** but for another device. The data are more noisy because of the contact problem of either V_2 or V_5 probe of this device, but the values of the data are almost same as those of **a, b, c**. Since we could have applied up to 6.4 V for this device during the Hall measurement, we used these data for $n_\square$ discussed in the main text.

temperature and the magnetic field control were done by using the Quantum Design physical properties measurement system (PPMS), where the sample was mounted on the Multi-Function Probe with Al wires attached to the electrode pads of the device by an ultrasonic bonding.

The voltage difference ΔV between the voltage probes V_2 and V_5 (Fig. 2d of the main text) perpendicular to the channel current I_{SD} was measured in the magnetic field B applied perpendicular to the channel surface. The jagged solid line (red) in Fig. S4a corresponds to the $\Delta V = V_5 - V_2$ for $V_G = 2.8$ V and $V_{SD} = 0.5$ V (left axis) and the smooth solid line (blue) corresponds to the magnetic field B (right axis), plotted against the time elapsed. Field independent contribution as indicated by the dashed line (green) in Fig. S4a, was subtracted from ΔV to obtain V_H plotted against B in Fig. S4b.

In general, I_{SD} is fixed during Hall effect measurements, however, in our measurement, it was not tractable because the current is not a proper valuable for the measurement of the highly resistive channel. Thus, we fixed V_{SD} instead of I_{SD} Despite the change of I_{SD} from time to time, our Hall resistance $R_{xy} = V_H / I_{SD}$ (Fig. S4c) showed an almost linear dependence of B for V_G in the range of 1–3 V. Hall coefficient $R_H = V_H / I_{SD} B$ was then obtained directly from the slope of the R_{xy}-B plot by a least-squares linear fitting of the data. The sheet carrier density $n_\square$ is deduced from $n_\square = 1/e\,R_H$.

The multi-terminal device used for the measurement of Fig. S4a–c underwent an electric breakdown for V_G above 3 V, so we repeated the same measurement for another multi-terminal device. The results are shown in Fig. S4d–f. The data were more scattered for this sample, because the electric contact either of the V_2 or V_5 probe might not be good. However, the magnitude of the data were not so different from the previous data set. Since we have applied V_G up to 6.4 V for this device, $n_\square$ derived from these data is used in the main text.

HYSTERESIS

The channel current I_{SD} did not show hysteresis if the applied V_G was below the threshold voltage V_{th}. The definition of V_{th} is described in the main text. Figure S5 shows an example of I_{SD}-V_G characteristics measured for the multi-terminal device shown in Fig. 2d of the main text. When we increase V_G, at $V_G \simeq 5.5$ V, $\log_{10} I_{SD}$ vs. V_G curve shows a deviation from a linear behaviour. Thus, we can assume the threshold voltage V_{th} is around 5.5 V. Interestingly, for $V_G < 5$ V, there appears no hysteresis in the I_{SD}-V_G characteristics as depicted by solid circles connected by solid lines (green) of Fig. S5, but for $V_G > 5$ V, large hysteresis appears.

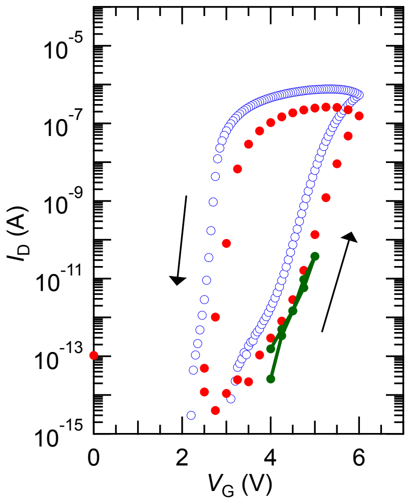

FIG. S5. I_{SD}-V_G curves of the multi-terminal FET device (Fig. 2d of the main text). Solid circles (red) were obtained by changing V_G from 0 V to 6 V and then to 0 V with the sweeping rate of 20 min/V (10 min waiting time for every 0.5 V step of V_G). Solid circles connected by solid lines (green) stand for the same I_{SD}-V_G but V_G was changed from 0 to 5 V. If the maximum V_G is below the threshold voltage V_{th} of around 5.5 V, no hysteresis appears. I_{SD}-V_G curve of the same device for the V_G sweeping rate of 30 min/V (3 min waiting time for every 0.1 V step of V_G) is depicted by the opaque circles (blue). It seems that I_{SD} increased about a decade, or rather that V_G decreased about 0.5 V.

It should be noted that this V_{th}, which is eventually identical to the threshold for the appearance of the hysteresis, is much larger than V_G at which the negative capacitance becomes dominant (see Fig. 3a in the main text). In other word, the observed negative capacitance, *i.e.*, the enhancement of $n_\square$ above $C_\square^{ins} V_G / e$, does not necessary accompany the hysteresis, which is generally attributed to an appearance of ferroelectricity in gate insulator. Indeed, as shown in the inset of Fig. 4a of the main text, the capacitance of our Parylene-C/HfO_2 gate insulator does not change more than 2 % even for the application of $V_G = 8$ V for about an hour.

Since the $SrTiO_3$ single crystal of 0.5 mm thick used in this work was a good insulator, we were necessary to apply V_G for sufficiently long time (about an hour) in order to accumulate the thermal carriers from the bulk substrate to the channel surface. Therefore, the longer time we take for increasing V_G, the larger I_{SD} we observe. The opaque circles (blue) in Fig. S5 correspond to I_{SD} as a function of V_G obtained by varing V_G in 50 % longer time. The I_{SD}-V_G curve shifted to smaller V_G direction (about 0.5 V) and to larger I_{SD} direction (about a decade). The shape of the I_{SD}-V_G hysteresis does not change much (for applying V_G smaller than V_{th}, no hysteresis appears in this case as well), and the result of the Hall effect measurement is not sensitive to I_{SD}. However, we believe

the origin of the hysteresis is by and large related to the unusual enhancement of $n_\square$, and should be unravelled by further experiments.

MOBILITY

Mobility μ of the carriers is given by

$$\mu = \frac{1}{e} \times \frac{\sigma_\square}{n_\square} ,$$

where e is the elementary charge and $\sigma_\square$ is the sheet conductance (without magnetic field B) defined as $\sigma_\square \equiv (I_{\rm SD}/W)/(V_{\rm SD}/L)$. For our multi-terminal FET device (Fig. 2d of the main text), $V_{\rm SD}/L$ is replaced by $\Delta V/\Delta L$, where $\Delta V \equiv V_3 - V_1$ and ΔL is the distance between V_3 and V_1 probes, *i.e.*, 12 μm. During the Hall effect experiments, we measured both $n_\square$ and $\sigma_\square$; the values of $\sigma_\square$ for $B=0$ are plotted against $n_\square$ as shown in Fig. S6. The solid line (green) is the least-squares linear fit, which gives $\mu = 10.9\,{\rm cm}^2/{\rm Vs}$ at room temperature.

In general, the field-effect mobility $\mu_{\rm FE}$ defined as

$$\mu_{\rm FE} \equiv \frac{1}{e} \times \frac{d\sigma_\square}{dn_\square} = \frac{\Delta L}{W} \frac{d}{d(C_\square V_{\rm G})} \left(\frac{I_{\rm SD}}{\Delta V}\right)$$

gives a good measure to evaluate the quality of the channel. Actually, if both $C_\square$ and ΔV are independent of $V_{\rm G}$, the above expression of $\mu_{\rm FE}$ becomes a well-know equation:

$$\mu_{\rm FE} = \frac{\Delta L}{C_\square W \Delta V} \frac{d I_{\rm SD}}{d V_{\rm G}} . \qquad ({\rm S1})$$

But, this is not a valid equation for our FET, because both $C_\square$ and ΔV depends significantly on $V_{\rm G}$ in our case. Nevertheless, in order to have a brief comparison of $\mu_{\rm FE}$ in the subthreshold region of our three-terminal FETs, we may substitute $\log_{10} I_{\rm SD} = V_{\rm G}/S$ to Eq. S1 with the subthreshold swing $S = 172\,{\rm mV/decade}$, $\Delta L = L = 20\,\mu{\rm m}$, $W = 80\,\mu{\rm m}$, $\Delta V = V_{\rm SD} = 1\,{\rm V}$, and $C_\square = 0.28\,\mu{\rm F/cm}^2$. Then, we obtain $\mu_{\rm FE} = 1.9 \times 10^7\, I_{\rm SD}\ {\rm cm}^2/{\rm Vs}$. Since the channel current $I_{\rm SD}$ can reach to 10^{-7} A or larger values, $\mu_{\rm FE}$ of our three-terminal FET can be in the range of 1–10 cm^2/Vs. This is consistent to the carrier mobility $\mu = 10.9\,{\rm cm}^2/{\rm Vs}$ deduced from the $\sigma_\square = n_\square e\mu$ relationship.

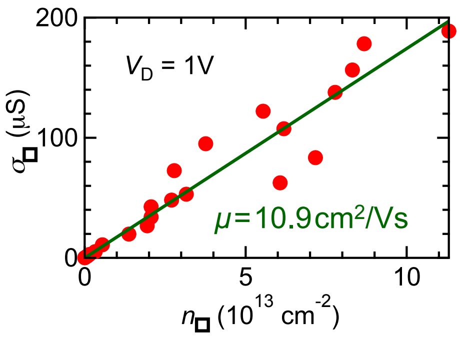

FIG. S6. Solid circles (red) correspond to the sheet conductivity $\sigma_\square$ of the channel of the multi-terminal FET device plotted against the sheet carrier density *nsq*; both were obtained during the Hall effect measurement, and all the values are for $B=0$. The carrier mobility $\mu = 10.9\,{\rm cm}^2\,{\rm V}^{-1}\,{\rm s}^{-1}$ was obtained by fitting the data with $\sigma_\square = e\mu n_\square$ (green solid line).

The observed value of $\mu \simeq 11\,{\rm cm}^2/{\rm Vs}$ is much larger than the values reported for other $SrTiO_3$ FETs,[5–8] corroborating the comparatively good quality channel of our FET. In addition, as a pilot work, we reported $\mu_{\rm FE} \sim 10\,{\rm cm}^2/{\rm Vs}$ using Ta_2O_5/Parylene-C bilayer on $SrTiO_3$ (Ref. 9). Both the thickness of Parylene-C ($\gtrsim$60 nm) and the channel area ($\gtrsim$0.16 mm^2) were one to four orders larger than those of the present work, hence the results are not simply compared. However, the results also support that the Parylene-C passivation of the $SrTiO_3$ surface is indeed significantly effective.

INHOMOGENEITY OF THE CHANNEL

Here we describe a phenomenological inhomogeneity model of the channel capacitance. Fig. S7a shows the $n_\square$ (open circles) and $1/C_\square^{\rm STO}$ (blue solid lines), both of which are same as in Fig. 4a in the main text. We have decomposed $1/C_\square^{\rm STO}$ in solid line (blue) into the dotted line (green) and the dashed line (purple); the former behaves $C_\square^{\rm STO} \to 20\,\mu{\rm F/cm}^2$ and the latter does $C_\square^{\rm STO} \to -30\,\mu{\rm F/cm}^2$, respectively, for $V_{\rm G} \to \infty$ (Inset of Fig. S7a). We have assumed here the channel is a *parallel* connection of the two capacitors, $C_\square^{\rm STO} = (1-\xi)\,C_\square^+ + \xi\, C_\square^-$, where $C_\square^-$ is the sheet capacitance of the domain schematically depicted as purple circles in Fig. S7b, and $C_\square^+$ is that of the background. ξ is determined to fit to $1/C_\square^{\rm STO}$ for each value of $V_{\rm G}$. In fact, the set of $C_\square^+$ and $C_\square^-$ can be arbitrary chosen, though either of them should be negative. Here, $C_\square^- = -30\,\mu{\rm F/cm}^2$ gives $\Delta\mu \simeq 24\,{\rm meV}$ for $\Delta n_\square \simeq 5 \times 10^{13}\,{\rm cm}^{-2}$ ($4 \lesssim V_{\rm G} \lesssim 6$), and $C_\square^+ = 20\,\mu{\rm F/cm}^2$ gives the thickness of 14 nm for the dielectric constant 310 of $SrTiO_3$. Those values are more realistic than $\Delta\mu \simeq -26\,{\rm eV}$ deduced from $C_\square^{\rm STO} = -0.31\,\mu{\rm F/cm}^2$ that we discussed above.

We think $1/C_\square^{\rm STO}$ may go to zero for $V_{\rm G} \to \infty$, but we could not measure $n_\square$ for such large $V_{\rm G}$; hence, in this work, we assume $C_\square^{\rm STO} \to -0.31\,\mu{\rm F/cm}^2$, leading to $\xi = 40\,\%$ for $V_{\rm G} \to \infty$. The domains with the negative capacitance must be metallic, while the domains with the positive

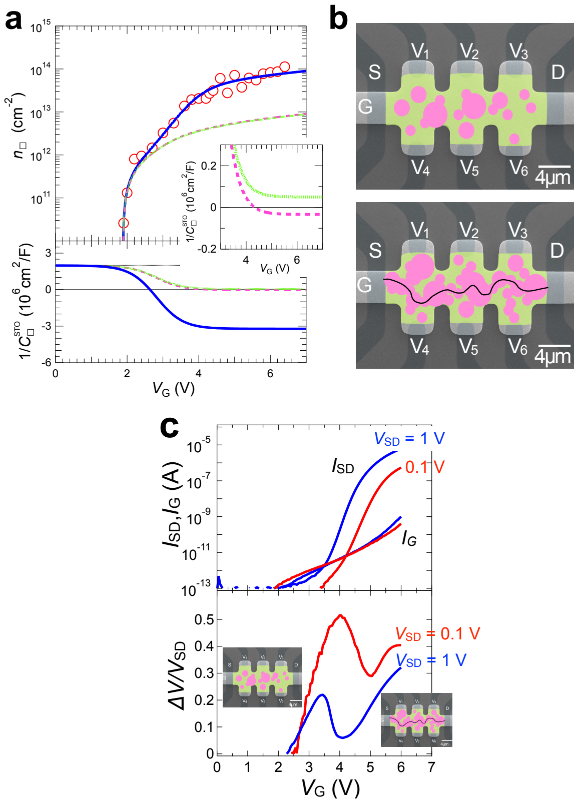

FIG. S7. Interpretation of $n_\square$ enhancement by a negative κ + inhomogeneity model. **a,** The solid lines (blue) are same as that of Fig. 4a in the main text, which are decomposed into $C_\square^+$ (dotted line in green) and $C_\square^-$ (dashed line in purple). $C_\square^+ \to 20\,\mu\mathrm{F/cm}^2$ while $C_\square^- \to -30\,\mu\mathrm{F/cm}^2$. **b,** The SEM picture of a FET device used for the Hall effect measurement ($L = 20$, $W = 4$ and the distance between V_1 and V_3 electrodes is $12\,\mu$m). The domain-formation is schematically depicted. Top: each circle (pink) corresponds to a metallic island with $C_\square^{\mathrm{STO}} = C_\square^-$ while the background (green) is less conductive with $C_\square^{\mathrm{STO}} = C_\square^+$. Bottom: by increasing V_{G}, metallic domains become larger and finally the current path (solid line in black) is formed. **c,** I_{SD} and I_{G} as well as $\Delta V \equiv V_3 - V_1$ normalised by V_{SD} plotted against V_{G}. In general, ΔV is expected to increase monotonously as a function of V_{G}, but the sudden decrease of ΔV while increasing V_{G} suggests the formation of the metallic path in the channel.

capacitance cannot be regarded as metallic, because $C_\square^+ = 20\,\mu\mathrm{F/cm}^2$ is not large enough. Hence, $\xi \to 40\,\%$ for $V_{\mathrm{G}} \to \infty$ means the source and drain electrodes are connected by a metallic path of connected $C_\square^-$ regions in the $C_\square^+$ background at a particular V_{G}. At that V_{G}, the potential distribution in the channel should be decreased rapidly.[9] This is indeed observed as shown in Fig. S7c; an evidence that the inhomogeneity plays a significant role in our FET.

However, if there exists such an inhomogeneity in the channel, what did we actually measure by the Hall effect? The problem of the Hall coefficient of inhomogeneous two-dimensional systems was intensively discussed in 1980s. A well-known example of the calculation of Hall coefficients of two-dimensional metal/insulator inhomogeneous system was given by D. Stroud and D. J. Bergman[10]. They concluded that the Hall coefficient of the 2D metal/insulator inhomogeneous system does not depend on the volume fraction of the metallic/insulating domains and is equal to the Hall coefficient of the whole homogeneous metallic plane. According to this calculation, the Hall coefficient observed by our measurement should be equal to that of the metallic domains, even if there is an inhomogeneity. This means the metallic domains have $C_\square^{\mathrm{STO}} = -0.31\,\mu\mathrm{F/cm}^2$ and $\Delta\mu \simeq -26\,\mathrm{eV}$, which is too large and would not be simply accepted.

There has been no theoretical model of Hall coefficient of the inhomogeneous two-dimensional system with negative capacitance. We hope our observation shown in this work will motivate further studies and substantiating results will be obtained in the future works.

[1] W. J. Gorham, *J. Polym. Sci.: Part A-1* **1966**, *4*, 3027.
[2] E. Meng, P. -Y. Li, Y. -C. Tai, *J. Micromech. Microeng.* **2008**, *18*, 045004.
[3] K. Arai, F. Yanagawa, S. Kurosawa, *J. Vac. Sci. Technol. B* **1984**, *2*, 658.
[4] T. Zaki, S. Scheinert, I. Hörselmann, R. Rödel, F. Letzkus, H. Richter, U. Zschieschang, H. Klauk, J. N. Burghartz, *IEEE Trans. Electr. Dev.* **2014**, *61*, 98.
[5] K. Shibuya, T. Ohnishi, T. Sato, M. Lippmaa, *J. Appl. Phys.* **2007**, *102*, 083713.
[6] T. Sato, K. Shibuya, T. Ohnishi, K. Nishio, M. Lippmaa, *Jpn. J. Appl. Phys.* **2007**, *46*, L515.
[7] H. Ohta, Y. Masuoka, R. Asahi, T. Kato, Y. Ikuhara, K. Nomura, H. Hosono, *Appl. Phys. Lett.* **2009**, *95*, 113505.
[8] Z. Zhu, Z. Luo, J. Xu, H. Zhao, S. Chen, *IEEE Electr. Dev. Lett.* **2013**, *34*, 927.
[9] A. B. Eyvazov, I. H. Inoue, P. Stoliar, M. J. Rozenberg, C. Panagopoulos, *Sci. Rep.* **2013**, *3*, 1721.
[10] D. Stroud, D. J. Bergman, *Phys. Rev. B* **1984**, *30*, 447.
